# Characterization of *Plasmodium vivax*-associated admissions to reference hospitals in Brazil and India

**DOI:** 10.1186/s12916-015-0302-y

**Published:** 2015-03-20

**Authors:** André M Siqueira, Marcus VG Lacerda, Belisa M L Magalhães, Maria PG Mourão, Gisely C Melo, Márcia AA Alexandre, Maria GC Alecrim, Dhanpat Kochar, Sanjay Kochar, Abhishek Kochar, Kailash Nayak, Hernando del Portillo, Caterina Guinovart, Pedro Alonso, Quique Bassat

**Affiliations:** Programa de Pós-graduação em Medicina Tropical, Universidade do Estado do Amazonas, Av. Pedro Teixeira, 25, Manaus, AM 69040-000 Brazil; Gerência de Malaria, Fundação de Medicina Tropical Dr Heitor Vieira Dourado, Av. Pedro Teixeira, 25, Manaus, AM 69040-000 Brazil; Instituto Nacional de Infectologia Evandro Chagas, Fundação Oswaldo Cruz, Av. Brasil, 4365, Rio de Janeiro, RJ 21040-360 Brazil; Centro de Pesquisas Leônidas e Maria Deane, Fundação Oswaldo Cruz, Rua Teresina, 476, Manaus, AM 69027-070 Brazil; Sardar Patel Medical College, SP Medical College Road, PBM Hospital, Bikaner, Rajasthan 334001 India; ISGlobal, Barcelona Institute for Global Health, Hospital Clínic - Universitat de Barcelona, Carrer Rosselló 132, 5°2ªE, 08036 Barcelona, Spain; Institució Catalana de Recerca i Estudis Avançats (ICREA), Passeig Lluís Companys, 23, 08010 Barcelona, Spain; Centro de Investigação em Saúde de Manhiça (CISM), Vila da Manhiça, Bairro Cambeve, Rua 12, Distrito da Manhiça, 1929 Maputo Mozambique

**Keywords:** *Plasmodium vivax*, Severe malaria, Clinical complications, India, Brazil

## Abstract

**Background:**

The benign character formerly attributed to *Plasmodium vivax* infection has been dismantled by the increasing number of reports of severe disease associated with infection with this parasite, prompting the need for more thorough and comprehensive characterization of the spectrum of resulting clinical complications. Endemic areas exhibit wide variations regarding severe disease frequency. This study, conducted simultaneously in Brazil and India, constitutes, to our knowledge, the first multisite study focused on clinical characterization of *P. vivax* severe disease.

**Methods:**

Patients admitted with *P. vivax* mono-infection at reference centers in Manaus (Amazon - Brazil) and Bikaner (Rajasthan - India), where *P. vivax* predominates, were submitted to standard thorough clinical and laboratory evaluations in order to characterize clinical manifestations and identify concurrent co-morbidities.

**Results:**

In total, 778 patients (88.0% above 12 years old) were hospitalized at clinical discretion with PCR-confirmed *P. vivax* mono-infection (316 in Manaus and 462 in Bikaner), of which 197 (25.3%) presented at least one severity criterion as defined by the World Health Organization (2010). Hyperlactatemia, respiratory distress, hypoglycemia, and disseminated intravascular coagulation were more frequent in Manaus. Noteworthy, pregnancy status was associated as a risk factor for severe disease (OR = 2.03; 95% CI = 1.2-3.4; *P* = 0.007). The overall case fatality rate was 0.3/1,000 cases in Manaus and 6.1/1,000 cases in Bikaner, with all deaths occurring among patients fulfilling at least one severity criterion. Within this subgroup, case fatality rates increased respectively to 7.5% in Manaus and 4.4% in Bikaner.

**Conclusion:**

*P. vivax*-associated severity is not negligible, and although lethality observed for complicated cases was similar, the overall fatality rate was about 20-fold higher in India compared to Brazil, highlighting the variability observed in different settings. Our observations highlight that pregnant women and patients with co-morbidities need special attention when infected by this parasite due to higher risk of complications.

**Electronic supplementary material:**

The online version of this article (doi:10.1186/s12916-015-0302-y) contains supplementary material, which is available to authorized users.

## Background

Malaria is a major public health problem in most tropical regions, and despite impressive reductions in the number of cases and deaths in the last decades, malaria resulted in an estimated 207 million episodes and 627,000 deaths in 2012 [1]. Although the attention of the scientific community has focused in past decades on the burden and consequences of *P. falciparum* infections [2,3], in recent years there has been a renewed interest in *P. vivax*-associated morbidity, as a result of the increasing report of its potential hazards to health [4-7].

*P. vivax* is the most geographically widespread parasite causing malaria in humans, with over 2.5 billion individuals exposed to the risk of infection; it is the predominant species in Latin America and some areas of the Asian and Pacific regions [1,8]. In a context of renewed and intensified commitment of the international community towards malaria eradication [4,9,10], a surge of interest has emerged regarding this species, possibly because of the major challenges posed for its control, ensuing from its poorly understood ability to present clinical relapses [5,10], but also due to its capacity to cause a more complicated disease [11-13]. *P. vivax* benign clinical course has now been completely reconsidered, as the evidence of life-threatening disease has been reported from diverse endemic areas, such as Latin America [7,14,15], India [16,17], and Southeast Asia and the Pacific regions [18,19]. In spite of the World Health Organization (WHO) recognition as early as 2006 of the potential of *P. vivax* to cause severe disease [20], there are still several gaps in knowledge to be filled [5,11,21,22].

The diverse range and rate of occurrence of clinical complications associated to *P. vivax* infection widely differ according to reports from different endemic regions, suggesting that many unaccounted factors, including those related to the parasite, the host, or the context in which the infection is produced may interact in causing the clinical presentation [12]. Indeed not all endemic areas have been reporting severity attributed to infection with this parasite, such as the Thailand-Myanmar border, where severity was reported only very recently [23]*.* In the absence of adequate experimental models, or the paucity of postmortem samples to further elucidate the pathophysiology of this infection [5,13,24], systematic multisite clinical studies become a unique opportunity to better understand this geographic variability and characterize the severe vivax malaria syndrome. Indeed, comprehensive reviews of the literature demonstrate that severe vivax malaria occurrence is an old, and not infrequent, phenomenon that was not being properly recognized [12,25,26].

We have used a common protocol in order to prospectively follow vivax malaria patients admitted to two distinct reference centers located in Brazil and India, aiming to comprehensively characterize and compare the clinical complications of *P. vivax* infection.

## Methods

### Study sites

**Figure 1:**
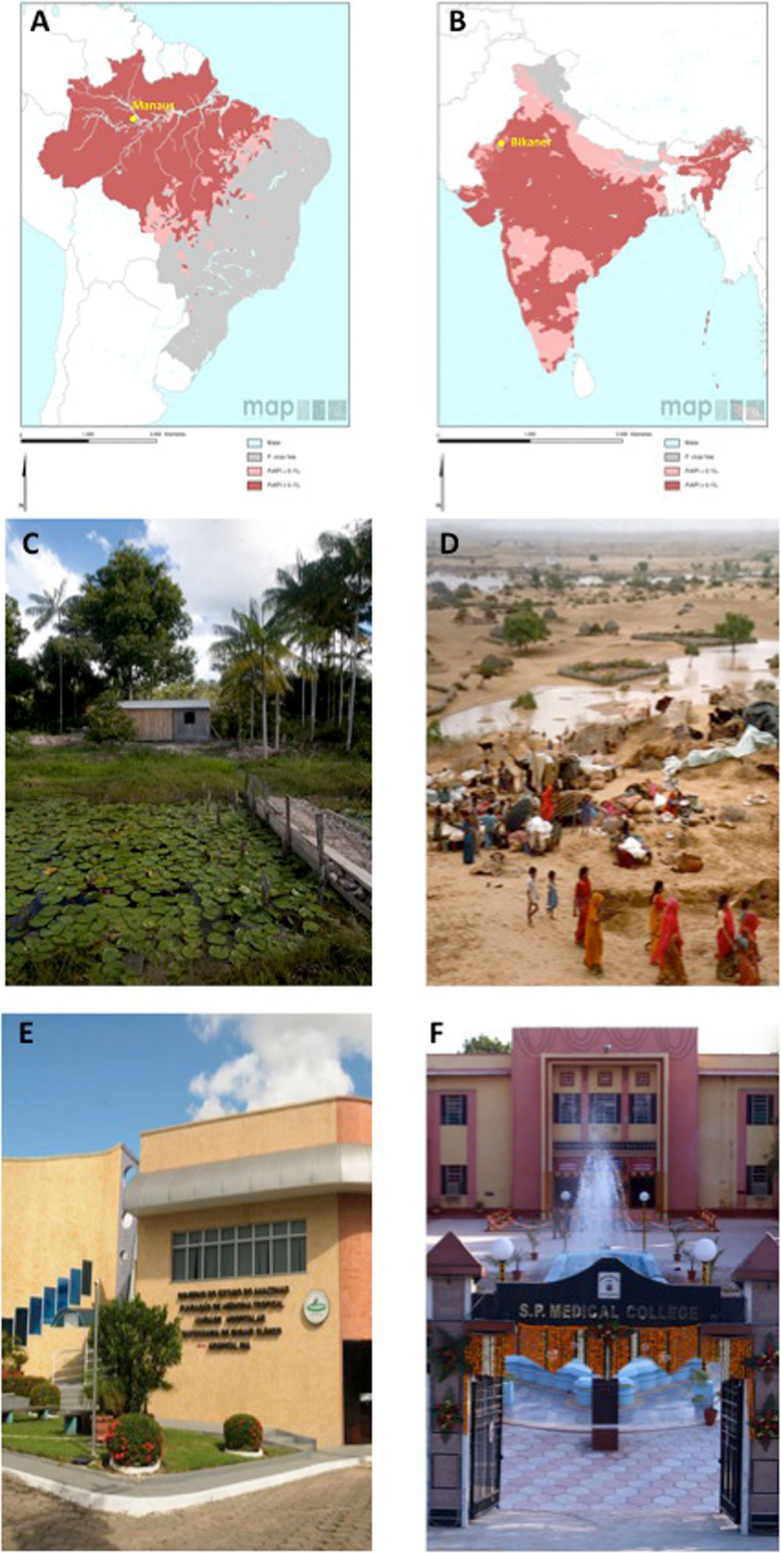
**Epidemiological profile of the two studied sites:** ***P. vivax*** **2010 annual parasite index in Brazil (A) and India (B) (reproduced from the Malaria Atlas Project** [8]**); malaria transmission scenarios in the rainforest, Manaus, Brazil (C) and in the desert, Bikaner, India (D); reference tertiary care center façades in Manaus (E) and Bikaner (F).**

The enrollment of cases was performed at two different reference tertiary hospitals located in Brazil and India. *Fundação de Medicina Tropical Dr Heitor Vieira Dourado* (FMT-HVD) is located in the city of Manaus, in the Brazilian Western Amazon, with 150 beds for hospitalization. This hospital is the reference tropical medicine and infectious disease center for the area, and receives patients from all over the Amazonas state, referred or not to this service. Admission is free of charge, and admission criteria for malaria patients are any clinical complications suggestive of severe malaria, or complications impacting proper antimalarial treatment, such as severe vomiting. Six other hospitals in town are the reference for pediatric diseases. *Anopheles darlingi* is the major malaria vector in the region, with transmission occurring continuously throughout the year with peaks in the beginning of the dry and the wet season. In recent years there has been a decrease in the number of reported episodes paralleled to an increase in the proportion of cases attributed to *P. vivax,* which is now responsible for more than 90% of cases of malaria in the region [27]. *Sardar Patel Medical College* (SPMC) hospital is located in Bikaner in the Rajasthan state of India, where, as a result of environmental and climatic reasons, malaria transmission has a well-defined seasonal pattern with practically no cases being reported during the dry season, and *P. vivax* is responsible for 50% of the cases [28]. *An. stephensi*, *An. culifascies*, and *An. annularis* are the major malaria vectors in the region (Figure 1). The hospital includes around 500 beds for hospitalization in general, and a specific malaria ward, which remains closed except for the duration of the malaria season. The hospital receives referrals from the entire Rajasthan state, although other hospitals (including private ones) are also available for patients in the area. Admission is free with the exception of certain subsidized procedures, and during the malaria season most of the patients are routinely admitted to this ward, often because they are being referred. Neither of the two hospitals is a specifically pediatric referral center, although pediatric patients may be admitted in both.

### Study design and patient selection

This was a hospital-based study of patients admitted with vivax malaria at the two study centers, regional references mostly for the adult population. Enrollment in Manaus occurred in two distinct periods (between April 2009 and March 2010, and between January 2011 and December 2011), while in Bikaner patients were recruited between August 2009 and October 2010. All initial microscopy-confirmed *P. vivax* infections (or patients with negative thick blood smears (TBSs) but with history of previous recent diagnosis in use of antimalarials) requiring admission, at clinician’s discretion (for example, for the presence of jaundice, thrombocytopenia, bleeding, vomiting, diarrhea, abdominal pain, non-lithiasic cholecystitis, spleen rupture, or overall compromised clinical status), regardless of age, were eligible for enrollment provided patients or their legal representatives signed an informed consent. All enrolled patients were hospitalized in separate wards designed for clinical research and were followed and evaluated daily until discharge, when the final outcome of each case was registered after consensual discussion performed by the clinical team involved in the study.

The same protocol for clinical and laboratory investigations was applied at both sites, including standard operating procedures (SOPs) and questionnaires. The study procedures were standardized and supervised by co-investigators from both sites and supervised by study monitors from ISGlobal (authors QB and CG) to minimize potential discrepancies in their application.

### Clinical evaluation and management

At enrollment all patients underwent an initial comprehensive clinical assessment based on a clinical history and physical examination. Data were prospectively collected using a standardized questionnaire, which included the description of the duration and intensity of the clinical symptoms, a thorough clinical examination, and complementary tests (complete blood cell count and biochemical analyses). The history of previous malaria infections and antimalarial use was obtained through medical history and health surveillance and health unit records revision. Blood samples were collected for PCR confirmation of *P. vivax* mono-infection and for a detailed evaluation of coexisting acute and chronic morbidities, when suspected by the clinicians, while women of reproductive age were additionally tested routinely for their pregnancy status. When available at each site, and according to individual clinical presentation, imaging exams were performed, such as abdominal or trans-vaginal ultrasound, computerized tomography, and fundoscopy. Treatment was provided according to WHO recommendations and national guidelines, with the use of chloroquine (25 mg/kg over 3 days) in non-complicated patients, or parenteral artemether or artesunate in patients with suspicion of severe malaria or severe vomiting, followed by primaquine (3.5 mg/kg over 7 days in Brazil, and over 14 days in India) [29,30]. In India, patients with severe criteria were also systematically prescribed wide spectrum antibiotics upon admission. Clinical assessments and TBS were performed on a daily basis. Additional and follow-up laboratory tests were performed at the physician’s discretion.

### Diagnosis of malaria

For the diagnosis and quantification of parasitemia, the TBS was prepared as recommended by the Walker technique [31]. In addition to the reading performed for patients’ diagnosis, each blood slide was analyzed independently by two microscopists. A slide was recorded as negative if no parasite was detected in the 200-field reading. If parasites were detected on the slide, quantification was performed by counting the number of asexual and sexual parasites until either 500 leukocytes or 500 parasites were counted (whichever occurred first). In case of discordance (species-specific, or in the density quantification whenever discrepancy was higher than 10%), a third reading was performed by a different microscopist. The parasite density was calculated by the arithmetic mean of two concordant readings and the white blood cell count obtained from the total blood count analysis as previously described [32]. In addition, species-specific real-time PCR for *Plasmodium* was performed to confirm *P. vivax* mono-infection status and exclude *P. falciparum* co-infection. *P. malariae* was not tested considering previous data from both sites confirming the absence of circulation of this species in the study areas. The extraction of total DNA from whole blood was performed using the QIAamp DNA 144 Blood Mini Kit® (Qiagen, USA), according to the manufacturer’s protocol, and amplification was done in an Applied Biosystems 7500 Fast System® using primers and TaqMan fluorescence labeled probes [33].

### Case definitions and classification

Patients were classified in relation to their clinical symptomatology and laboratory results. The presence of the WHO-defined severe malaria criteria and syndromes was systematically assessed at admittance and during the whole length of hospitalization at both sites, with cases being classified accordingly to the criteria described in Table 1 [30]. The results for the worst tests were recorded in the Case Report Form (CRF) and the last results were used for case closure purposes. The systematic assessment at admission consisted of full blood count, biochemistry analyses (creatinine, urea, bilirubin, aspartate aminotransferase, alanine aminotransferase, gamma-glutamyltransferase, albumin, and venous lactate), urine analysis, pregnancy tests for women between 12 and 50 years of age, and chest X-rays. We also classified patients according to the number of severe criteria fulfilled as a proxy of higher severity, for which more specific and easier applicable criteria [7] were used: severe anemia (hemoglobin <7 g/dL in adults and <5 g/dL in children); respiratory distress/acute lung injury; circulatory shock (systolic blood pressure < 90 mmHg refractory to fluid); acute renal failure; and cerebral malaria. A cut-off was decided to categorize cases as more severe if they presented three or more severe criteria.

**Table 1:** **Demographic and epidemiological characteristics of enrolled patients at both sites**

	**Severe**	**Non-severe**	***P***
	**N = 197**	**N = 518**	
Female sex	133 (55.5%)	225 (43.5%)	0.003
Age < 12 years old	10 (5.1%)	23 (5.0%)	0.422
Duration of fever (days): Mean (SD)	5.4 (3.8)	5.7 (7.1)	0.453*
First malarial infection	27 (12.7%)	88 (16.5%)	0.187
Antimalarials in the preceding 30 days	41 (18.6%)	78 (14.2%)	0.030
Geometric mean *P. vivax* density on admission (parasites/mm^3^; 95% CI)^¶^	2,267 (1,872-2,745)	1,902 (1,667-2,169)	<0.001
Pregnancy (among females of reproductive age)	37/114 (32.5%)	97/227 (18.5%)	0.004
Any co-morbidity	31 (15.9%)	92 (17.8%)	0.527
Heart rate (beats/min): Mean (SD)	87.1 (12.8)	85.0 (11.8)	0.032*
Respiratory rate (breaths/min): Mean (SD)	18.8 (4.0)	19.4 (4.9)	0.126*
Temperature (°C): Mean (SD)	37.9 (1.1)	37.5 (1.1)	<0.001*
Hepatomegaly	47 (21.6%)	141 (25.8%)	0.217
Splenomegaly	68 (31.2%)	131 (24.0%)	0.041
Hemoglobin (g/dL): Mean (SD)	7.7 (3.1)	10.9 (2.6)	<0.001*
White blood cell count (× 1,000 cells/mm^3^): Mean (SD)	8.6 (10.4)	6.5 (3.8)	<0.001*
Platelets (× 1,000 cells/mm^3^): Mean (SD)	74.1 (88.9)	72.5 (90.9)	0.824*
Creatinine (mg/dL): Mean (SD)	1.4 (1.6)	0.9 (0.4)	<0.001*
Urea (mg/dL) : Mean (SD)	51.2 (49.7)	38.9 (20.3)	<0.001*
Total bilirubin (mg/dL): Mean (SD)	2.9 (5.9)	2.2 (3.5)	0.0498*
ALT (U/L): Mean (SD)	50.5 (85.6)	42.2 (81.3)	0.222*
Lactate (mmol/L): Mean (SD)	5.0 (13.4)	2.0 (2.5)	0.010*

*P*-value obtained by chi-square test or Student *t* test with the latter identified (*); ^¶^restricted to patients without prior exposure to antimalarials (*P*-value derived from Poisson regression).

The performance of additional investigations for co-morbidities was triggered either for specific manifestations and/or at the physician’s discretion. These criteria included: i) severe anemia: hemoglobinopathies investigation and glucose-6-phosphate dehydrogenase (G6PD) assessments, and abdominal ultrasound; ii) respiratory distress: chest X-rays, blood gas, chest computerized tomography (CT) scan, and blood cultures; iii) ALT. 200 U/L: serology against hepatitis A, hepatitis E (only in India), and dengue; iv) creatinine > 3 mg/dL: renal ultrasound, 24-h proteinuria measurement and creatinine clearance; v) cerebral malaria: CT scan and lumbar puncture (if not contraindicated); and vi) severe abdominal pain: abdominal ultrasound. HIV testing (serology) was conducted for all admitted patients. Additionally, co-infection with dengue fever (by serology and PCR), and leptospirosis (serology) were systematically investigated in all admitted patients in Manaus. Blood cultures were performed in Bikaner, although samples were collected after patients had been started on antibiotics, a practice which is routine among patients with severity criteria. In Manaus, two blood culture samples were drawn for all admitted patients with severe criteria (10 mL of blood). Chronic co-morbidities were determined based on patients’ provided information and assessment of medical records. For these analyses, patients admitted due to primaquine-induced hemolysis (only observed in Manaus) were excluded, and the details concerning its presentation and outcomes will be presented in a separate publication (in preparation).

### Statistical analysis

All the data were double entered in forms designed in the Open Clinica® online platform, with the discordances and inconsistencies corrected according to a pre-established protocol. Only hospitalized patients with PCR-confirmed *P. vivax* mono-infection were included for the analyses. The demographic and clinical characteristics of patients were described in terms of proportions and compared using the chi-square or Fisher test as appropriate. Unadjusted simple logistic regression analyses were also performed considering different outcomes, especially the fulfillment of WHO severe criteria for *P. falaciparum* [20] and death. Further description and analyses were performed for each of the main severe complications observed in order to provide a broader picture of the clinical spectrum of this infection. Unadjusted linear regression was undertaken to explore the association between number of severe criteria and risk factors. Multiple analyses were not performed, because the sample size did not offer sufficient power for that purpose. The diagnostic performance of laboratorial parameters for identifying severity or multiplicity of severity criteria was assessed by means of receiver operating characteristic (ROC) curves. The analyses were performed using SPSS® version 16.0 for Windows (SPSS® Inc. Chicago, IL, USA) and Stata version 13.1 (StataCorp®, College Station, TX, USA).

### Ethical considerations

The study was approved by the Ethics Review Board (ERB) of *Hospital Clínic*, Barcelona, Spain (4510/2008), the ERB of *Fundação de Medicina Tropical Dr Heitor Vieira Dourado,* Manaus, Brazil (1943/2008), the National Brazilian Committee of Ethics (CONEP) (343/2009), and the ERB of *Sardar Patel Medical College & Associated Groups of Hospitals*, Bikaner, India (24/12/2008).

## Results

### Study subjects

**Figure 2:**
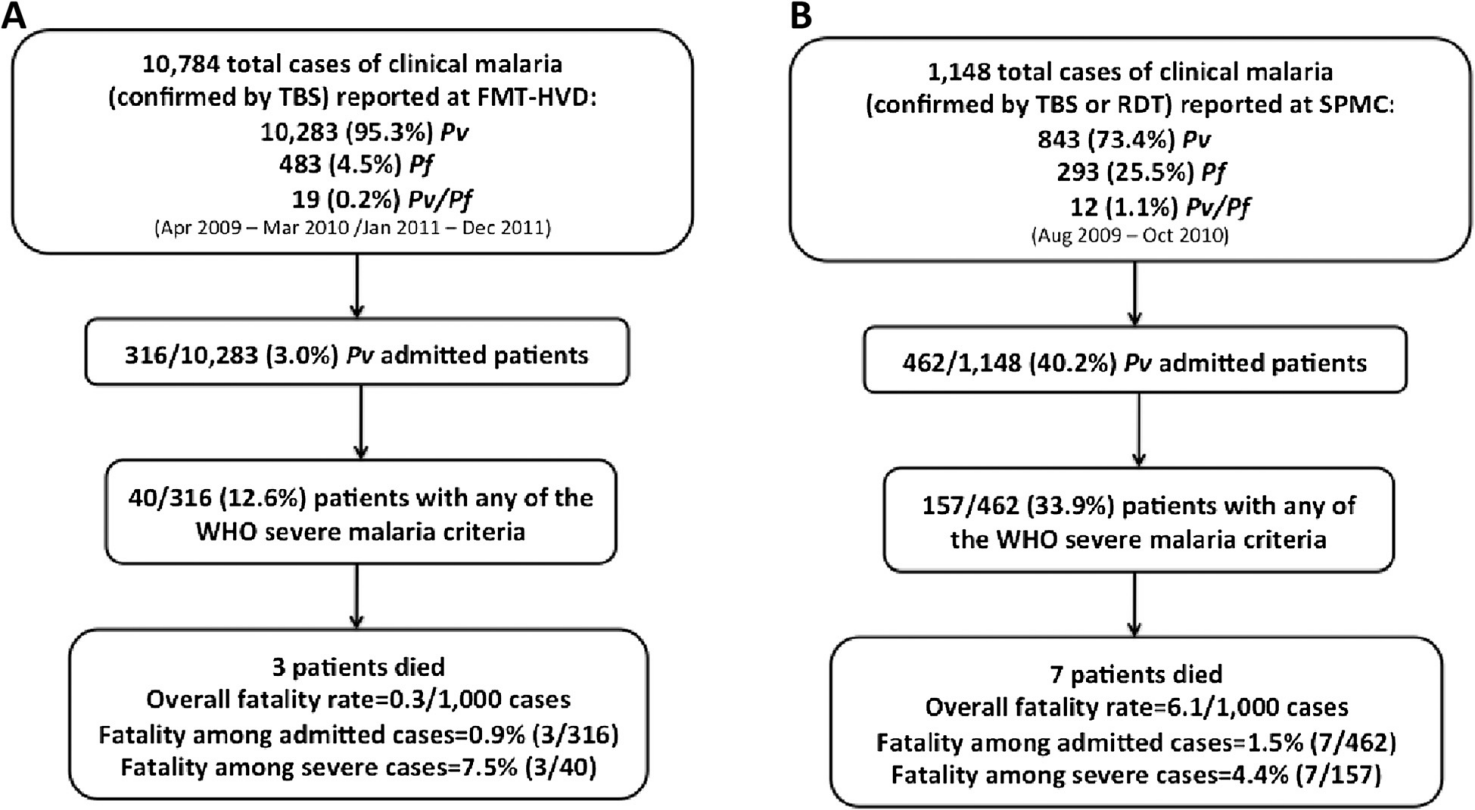
**Number of diagnosed, hospitalized, severe, and deceased patients with** ***P. vivax*** **malaria, during the study period in Manaus (A) and Bikaner (B).**

During the study period, 316 and 462 patients were admitted with vivax malaria in Manaus and Bikaner respectively (Figure 2 and Table 1) with three and seven associated fatalities per site. Among the patients with fatal outcome, there was a predominance of women (eight patients), four of them pregnant, with patients from Manaus presenting higher age and number of co-morbidities compared to Bikaner. The characteristics of the 10 patients with fatal outcome are presented in Additional file 1: Table S1. Only in Manaus was *P. falciparum/P. vivax* co-infection seen in one patient with severe criteria. Among patients without WHO criteria, no deaths were observed. On admission, 24 patients had a negative TBS, all of them having initiated antimalarials in other health units, of which PCR was positive for *P. vivax* in 14 of them. There were 47 patients admitted in Manaus due to primaquine-associated hemolysis and associated complications, all of them with G6PD deficiency, with no patient being diagnosed with this condition in Bikaner.

Regardless of the frequency of specific complications, in Manaus more patients with co-morbidities were admitted, however with similar proportion of deaths (Table 2). Antibiotics were administered to 24 patients in Manaus, for treatment of pneumonia in 16 patients, cellulitis in 4 patients, amebiasis in 2, and enteritis in 2. Although blood and urine cultures were obtained from all these patients prior to therapy, only in one patient was a microorganism identified (*Staphylococcus aureus* associated with cellulitis). In Bikaner, antibiotics were administered to 230 patients (with 62.7% of those with at least one severe criterion receiving them). Additional investigations were performed, as aforementioned, at physicians’ discretion or triggered by specific manifestations, limiting the estimation of its occurrence within the study population. The main chronic and acute co-morbidities diagnosed in these patients are described in Table 2. Dengue fever co-infection, due to its high occurrence and public health importance in Manaus, has been described in another publication [34].

**Table 2:** **Description of chronic and acute co-morbidities diagnosed in** ***P. vivax***-**infected patients admitted to two reference centers in Brazil and India**

	**n**
Chronic co-morbidities*	**84**
Arterial hypertension	34
Diabetes	28
Chronic hepatitis C infection^1^	14
HIV/AIDS^2^	8
Chronic hepatitis B infection^3^	8
Chronic renal failure	2
**Acute co-morbidities and investigations**	**87**
Blood culture	
Dengue fever^4^	68
Pneumonia	9
Enteric fever	3
Lepstopirosis^5^	2
Hepatitis A infection^6^	2
Varicella-zoster virus meningoencephalitis	1
Sickle cell disease crisis	1
Diphteria	1

^1^Defined by positive anti-HCV serology; ^2^defined by serology; ^3^defined by positive HBsAg; ^4^defined by positive PCR and/or NS1 test; ^5^defined by positive IgM; ^6^defined by positive IgM anti-HAV.

*Uncertain denominators as investigations were made at attending physician’s discretion.

### Clinical manifestations and severity

Patients presented a varied array of clinical complications associated with *P. vivax* mono-infection, including atypical complications seen only on imaging exams. Anemia was the more frequent complication at both sites, followed by acute renal failure. Respiratory distress, hyperlactatemia, hypoglycemia, and disseminated intravascular coagulation, although rare, were mostly seen in Manaus (Table 3). There was a higher proportion of patients presenting with three or more complications in Bikaner compared to Manaus (16.2% versus 10.7%, respectively; *P* = 0.063), with no further discrepancies observed between sites.

**Table 3:** **Epidemiological and clinical characteristics of** ***P. vivax*** **patients admitted to tertiary care centers in Brazil and India, with the diagnosis of severe malaria according to WHO criteria**

	**Manaus (Brazil)**	**Bikaner (India)**	***P***
**Demographic and epidemiological characteristics**			
Female sex	16/40 (40.0%)	98/157 (62.4%)	0.010
Age <12 years old	7/40 (17.5%)	3/157 (1.9%)	<0.001
First malarial infection	25/40 (62.5%)	149/150 (99.3%)	<0.001
Pregnancy (among females of reproductive age)	11/16 (68.8%)	67/98 (68.4%)	0.976
**Clinical characteristics**			
Severe anemia (Hb < 5 g/dl if age < 12 and Hb < 7 if age ≥ 12)	19/40 (47.5%)	83/157 (53.2%)	0.519
Acute renal failure (Creatinine > 3 mg/dL and/or urinary output < 0.5 ml/kg/h for 6 hours)	9/40 (22.5%)	18/157 (11.5%)	0.073*
Hyperlactatemia (lactate > 5 mmol/L)	5/40 (15.2%)	4/157 (2.6%)	0.343*
Respiratory distress (PaO_2_/FiO_2_ ≤ 300 mmHg and/or bilateral opacities on X-ray)	7/40 (17.5%)	5/157 (3.2%)	0.002*
Hypoglycemia (blood glucose < 40 mg/dL)	4/40 (10.0%)	1/157 (0.7%)	0.001*
Shock (SBP > 90 mmHg refractory to fluid resuscitation)	3/40 (7.6%)	7/157 (4.6%)	0.463*
DIC (prolonged aPTT, platelet < 50x10^9^/L and elevated FDP)	2/40 (5.0%)	0/157 (0%)	0.005*
Cerebral malaria (GCS ≤ 9 in adults or BCS ≤ 2 in children and/or repeated convulsions)	1/40 (2.5%)	5/157 (3.2%)	0.817*
Two or more complications	20/40 (50.0%)	75/157 (47.8%)	0.801
Three or more complications	5/40 (12.5%)	16/157 (10.2%)	0.673*
Presence of any other acute co-morbidity	6/40 (15.0%)	6/157 (0.6%)	<0.001*
Presence of any chronic co-morbidity	14/40 (35.0%)	4/157 (2.5%)	<0.001*
Death	3/40 (7.5%)	7/157 (4.4%)	0.434*

*P*-value obtained by chi-square test or Fisher test with the latter identified (*); Hb, hemoglobin; PaO_2_, partial pressure of arterial oxygen; FiO_2_, fraction of inspired oxygen; SBP, systolic blood pressure; DIC, disseminated intravascular coagulation; aPTT, activated partial thromboplastin time; FDP, fibrinogen degradation product; GCS, Glasgow Coma Scale; BCS, Blantyre Coma Scale.

For the outcome of presenting at least one of the WHO severe malaria criteria, there was association with female gender, first malarial infection, and pregnancy. For multiple severity criteria, the only variable showing evidence of association was the presence of chronic co-morbidity, with borderline evidence for an association with both pregnancy status and female gender. There was evidence of association of female gender as a risk factor for death among *P. vivax* admitted patients, and although the proportions of individuals with chronic co-morbidities and non-pregnant women were higher among the patients who died, the differences were not meaningful and the lack of power needs to be taken into consideration (Table 4). Similar findings resulted when the same analysis was performed per site (data not shown). Further characterization of specific manifestations is shown below. An additional linear regression analysis on the association with the number of severe criteria in each patient with the same investigated risk factors was also undertaken, with the only variables with significant association being female gender (coefficient = 0.45; *P* = 0.001), pregnancy status (coefficient = 0.81; *P* = 0.002), and the presence of any chronic co-morbidity (coefficient = 0.45; *P* = 0.049).

**Table 4:** **Univariate analysis of risk factors associated to WHO severe disease, three or more severity criteria, and death, in** ***P. vivax*** **patients admitted to two tertiary care centers in Brazil and India**

	**Severe n/N (%)**	**Non-severe n/N (%)**	**OR (95% CI)**	***P***	**Presence of three or more severe criteria n/N (%)**	**Absence of three or more severe criteria n/N (%)**	**OR (95% CI)**	***P***	**Dead n/N (%)**	**Survived n/N (%)**	**OR (95% CI)**	***P***
Female gender	114/197 (57.9)	230/518 (44.4)	1,7 (1.2-2.4)	0.001	14/21 (66.7)	330/694 (47.6)	1.7 (1.1-2.7)	0.022	8/10 (80.0)	336/705 (47.7)	2.6 (0.8-8.4)	0.119
Age < 12 years old	10/197 (5.1)	21/518 (4.0)	1.3 (0.6-2.9)	0.476	2/21 (9.5)	25/694 (3.6)	1.3 (0.4-4.5)	0.647	0/10 (0)	27/705 (3.8)	-	0.440
First malarial infection	174/190 (91.6)	431/501 (86.0)	1.8 (1.1-3.1)	0.044	3/20 (15.0)	83/671 (12.4)	1.0 (0.5-2.2)	0.725	7/10 (70.0)	598/681 (87.8%)	0.3 (0.1-1.3)	0.090
Pregnancy^¶^	36/114 (31.6)	40/230 (17.4)	2.3 (1.3-3.8)	0.003	8/14 (57.1)	65/330 (19.7)	3.2 (1.7-6.1)	<0.001	4/8 (50.0)	78/336 (23.3)	2.6 (0.7-10.1)	0.157
Peripheral parasitemia >5,110 parasites/mm^3^*	18/98 (18.4)	43/149 (28.9)	0.6 (0.3-1.0)	0.063	10/46 (21.7)	51/201 (25.4)	0.8 (0.4-1.8)	0.607	4/9 (44.4)	57/238 (24.0)	2.5 (0.7-9.8)	0.175
Duration of fever prior to admission >7 days	32/197 (16.5)	156/517 (30.3)	0.5 (0.3-0.8)	0.008	2/21 (12.0%)	157/694 (22.7)	0.5 (0.2-1.2)	0.098	1/10 (10.0)	184/705 (26.2)	0.4 (0.1-2.9)	0.329
Presence of any acute co-morbidity	7/197 (3.6)	31/518 (6.0)	0.7 (0.3-1.6)	0.439	4/21 (19.0)	38/694 (5.5)	1.3 (0.4-3.9)	0.648	2/10 (20.0)	38/705 (5.4)	5.3 (0.7-24.0)	0.084
Presence of any chronic co-morbidity	18/197 (9.1)	60/518 (11.6)	0.9 (0.5-1.4)	0.349	6/21 (28.6)	72/674 (10.4)	1.1 (0.5-2.0)	0.008	3/10 (30.0)	75/705 (10.6)	4.0 (1.2-13.3)	0.051*

^**¶**^Analysis restricted to women of reproductive age (12-50 years-old); *restricted to patients not in use of antimalarials at the initial assessment (5,110 parasites/mm^3^ was the lower limit of the highest quartile).

### Severe anemia

Severe anemia was the most common clinical complication at both sites, occurring among 102 (51.8%) of the admitted patients, with a higher proportion of females compared to males (18.6% versus 5.0%; *P* < 0.001), with more pregnant women presenting severe anemia (37.2% versus 19.1%; *P* = 0.001). Surprisingly, age, chronic co-morbidities, previous malaria history, and report of antimalarial use in the preceding 30 days were not associated with severe anemia. The proportion of patients presenting with jaundice, splenomegaly, and hepatomegaly did not differ between patients with and without severe anemia. No hemoglobinopathies were detected among our cohort, reflecting the low prevalence in both study areas. Blood transfusions were administered to 69 patients, corresponding to 67.6% of individuals fulfilling the severe anemia criteria, illustrating its effectiveness in identifying the most severe cases. Six patients with severe anemia experienced fatal outcomes (fatality rate = 6.0%).

### Acute renal failure

Acute kidney injury was characterized in a total of 27 patients from both sites, 26 above the age of 12. A similar proportion of both genders was affected, with only two pregnant women presenting this complication. The mean creatinine at presentation for these patients was 4.1 mg/dL (SD = 2.8), with 41.1% presenting abnormal renal ultrasound characterized by hyperechogenicity in the cortex, usually associated with interstitial nephritis. Dialysis therapy was undertaken in 11 (40.7%) of these patients, with a total of nine patients being admitted to the ICU and five patients with this complication dying (fatality rate = 18.5%).

### Respiratory distress

Respiratory distress in our series was observed with considerably higher frequency in Manaus (Table 3). Major changes in chest X-rays were observed for eight patients (66.7%), mainly characterized by bi-basal interstitial opacities. No children presented with acute respiratory distress syndrome (ARDS), and there was no association with gender or pregnancy status in its occurrence. There was, however, strong evidence of association of ARDS with having initiated antimalarials previously to admission. Six of the patients presented respiratory distress upon admission, which was characterized as acute lung injury, and of these five had begun antimalarials within the preceding 72 hours. Two patients developed ARDS as part of a systemic inflammatory response syndrome with multi-organ dysfunction and subsequent death. One hundred nineteen patients arrived with the previous diagnosis of *P. vivax* infection and use of chloroquine (with or without primaquine) prescribed at other health services. Considering the clinical presentation on admission, patients who used antimalarials in the preceding 72 hours were more likely to present with respiratory distress (OR = 10.7; 95% CI 2.1-55.9). Four patients with ARDS died, resulting in a fatality rate of 33.3%. Cover image of a patient presenting ARDS post-chloroquine initiation.

### Cerebral malaria

Cerebral malaria was characterized, following the WHO guidelines, as impaired conscience or repeated convulsions. It was more common in India, affecting five patients, while only one was diagnosed in Brazil (Table 3). Of the six patients with central nervous system syndromes, four were male, and their ages ranged from 17 to 75 years of age. All patients had impaired consciousness on admission, with one 22-year-old Indian female patient also presenting with repeated seizures. Lumbar puncture was performed on three patients without significant changes in any of them. In one male patient from Manaus, varicella-zoster virus was identified in the cerebrospinal fluid (there were no changes in the cerebrospinal fluid analysis). CT scans were performed for three patients without pathological findings.

### Other syndromes

Besides the aforementioned severity criteria, patients also presented with hyperlactatemia, hypoglycemia, and disseminated intravascular coagulation (Table 3). Furthermore, clinical complications outside the WHO severe criteria definitions were also characterized among the admitted patients, such as mild hepatitis (ALT > 200 u/mL) in 20 patients, acalculous cholecystitis in 9 patients, and splenic rupture or infarction in 3 patients. From the three cases with spleen-related complications, two were diagnosed with spleen rupture characterized by very low hemoglobin concentrations (4.7 mg/dL and 4.2 mg/dL) and spleen hematoma observed at the CT scan, while the patient with spleen infarction, as characterized by both abdominal ultrasound and CT scan, presented with intense upper right quadrant abdominal pain, with none of them presenting any other severe manifestation, such as circulatory shock. All of them were managed conservatively and recovered without further sequelae.

All pregnant women were submitted to obstetric ultrasound and appropriate testing, and were followed up to determine the pregnancy outcome through a parallel ongoing study protocol. Apart from one patient presenting subchorionic hematoma with subsequent abortion at 12 weeks of pregnancy, no other pregnancy-associated complications were observed on admission. Among the 36 pregnant women with severe manifestations, the most common complications were severe anemia (28), ARDS (6), cerebral malaria (2) and acute kidney injury (2). All four pregnant women who died presented with ARDS, with three of them also presenting with anemia and one of them also presenting with acute kidney injury and circulatory shock. Although no autopsies were performed, no additional causes of death were suggested.

### Discriminant performance

**Figure 3:**
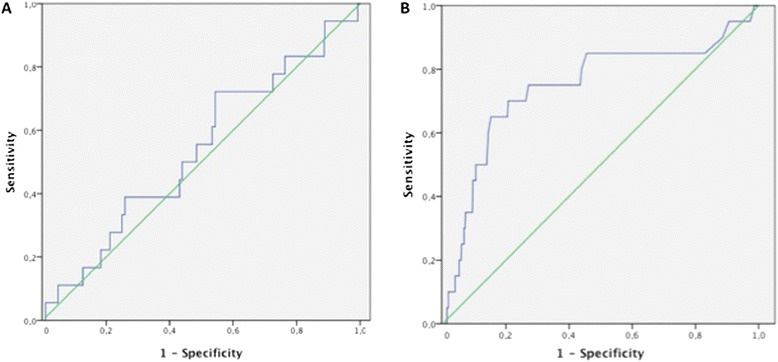
**ROC curves depicting the discriminatory performance of peripheral parasitemia before the use of antimalarials (A) (AUC = 0.538; 95% CI = 0.421-0.657;** ***P*** **> 0.05); and serum total bilirubin (B) (AUC = 0.743; 95% CI = 0.611-0.875;** ***P*** **< 0.001) for differentiating** ***P. vivax*** **-associated three or more criteria of severity.** AUC: area under the curve; green line: reference.

The performance of laboratorial tests (hematology, biochemistry, and parasitemia) to discriminate severe cases, deaths, or cases with multiple severity criteria was assessed using ROC curves. Except for total serum bilirubin, which presented a reasonable discriminative performance (AUC = 0.743; 95% CI = 0.611-0.875; *P* < 0.001), with values above 2.5 mg/d presenting a sensitivity of 47.0% and a specificity of 96.3%, resulting in an overall accuracy of 76.7% (Figure 3) no other parameters presented good accuracy for any of the evaluated outcomes. Parasite density among patients who had not received antimalarials prior to admission presented a very poor accuracy to discriminate patients with three or more severity criteria (AUC = 0.538; 95% CI = 0.421-0.657; *P* > 0.05), as did platelet counts and alanine aminotransferase (AUC = 0.417 and 0.499, respectively), indicating that these parameters should not be used as criteria to identify patients with *P. vivax*-associated complications.

## Discussion

This is, to our knowledge, the first multisite study conducted in two very distinct geographical areas, trying to comprehensively describe severe vivax malaria cases after an adequate diagnosis, with PCR confirmation of exclusive *P. vivax* mono-infection as an important strength. Through additional investigations, we have been able to identify a high number of associated co-morbidities, although by not performing a systematic investigation in all admitted cases from both sites, there was an important limitation on estimating the prevalence of their occurrence and association with severe syndromes, an important issue to consider [35,36]. Furthermore, although we have tried to ascertain the presence of previous chronic co-morbidities through medical interviews and patient record revisions, the possibility of underreporting and underdiagnosis must be considered. Indeed, one of the main concerns of the protocol was to exclude misdiagnosis and co-infection with *P. falciparum* by applying validated and standardized molecular methods [33], as microscopy can frequently miss out or even misdiagnose a varied proportion of cases. Our data provide yet again a robust confirmation of the potential of this species to cause significant morbidity and even mortality, a fact now widely recognized by the scientific community and by WHO in its recently published severe malaria monograph [37]. Indeed, the associated case fatality rate (CFR) among admitted patients to the study in the Brazilian Amazon (0.9%) and Indian Rajasthan (1.5%) is not negligible, and is comparable to CFRs previously described in Papua New Guinea [38] and Indonesia [18], and not too dissimilar to those for *P. falciparum* in Africa [2]. These findings must be taken cautiously, as the low incidence of *P. falciparum* in the study areas and the hospital-based design can limit comparisons and estimates of community CFRs. However, when compared the overall hospitalization and fatality rates, a striking difference could be observed between Manaus and Bikaner, as the latter presented much higher rates, up to 20-fold higher for fatality.

Conducting multisite studies allows for an active comparison of the different clinical complications and associated rates that would be expected by the very distinct demographic, socioeconomic characteristics, health systems features, and, especially, the local malaria transmission dynamics in the different *P. vivax* endemic areas. The influence of these factors could already be observed by comparing the descriptions of clinical epidemiology of complications associated with this infection from different sites, showing that in areas of higher transmission intensities, children are the most frequently affected population [18,19,39,40], while in areas of moderate and low intensities, adults contribute more to the proportion of severe cases [7,14,41]. Our study highlighted many of these differences. For instance, when comparing patients hospitalized at both sites, there was evidence that the higher proportion of patients being admitted in India was secondary to a higher frequency of first malarial infections in that location, reflecting distinctive transmission dynamics, including a differential pattern of relapse rates that could contribute to some degree of clinical immunity among Brazilian patients [42,43]. The spectrum of clinical complications in both sites was broadly similar, with severe anemia being by far the most frequent of the complications, as previously described in other *P. vivax* endemic settings [14,17,18,44], and followed by acute renal failure, a complication that seems to be particularly frequent in the two countries where the study was conducted albeit rare elsewhere [37]. The presence of three or more severity criteria was also frequent and common at both sites. The proportion of severe malaria cases with acute lung injury/respiratory distress was, however, significantly higher among Brazilian patients. This particular difference could be due to a number of factors, including a higher proportion of Indian patients being systematically treated with antibiotics, or differences in parasite virulence or host genetics, which could not be assessed in this study and should be considered in future studies. However, our data point to the beginning of antimalarial treatment as a triggering phenomenon of respiratory distress, which needs further investigation. There were only six episodes of cerebral malaria among our patients, with one of them presenting co-infection with varicella-zoster virus (VZV) isolated by PCR from the CSF, in agreement with data from other settings showing low rates of occurrence [45].

There were 10 deaths among our admitted patients’ series, and although there was low power for assessment of prognostic factors, there was evidence that female gender (probably confounded by the risk conferred by pregnancy) and chronic co-morbidities were associated with a higher risk of dying. One of the most striking findings in this study was the confirmation that pregnancy seems to be a clear risk factor for severe vivax malaria and even for death (despite being only borderline significant). It has been argued that, unlike *P. falciparum*, *P. vivax* rarely causes severe malaria in pregnant women [37,46-48]; however, our series confirms a very high and almost identical proportion (above 2/3 in both sites) of pregnancies among the cases of severe malaria occurring in women of reproductive age, and four maternal deaths (all occurring in Bikaner). Pregnant women were systematically evaluated for obstetric complications, allowing us to diagnose an abortion occurring as a consequence of a subchorionic hematoma which we attributed to a malaria-related complication, leading us to hypothesize that further studies aimed at this population could properly detect and estimate the pregnancy-related burden of *P. vivax* infection. We were also able to detect the frequent occurrence of pregnancy-related complications such as subchorionic hematoma. On the other hand, the histopathological evidence in the literature points to minor damage of *P. vivax*-infected placentas [49,50]. Ongoing studies specifically targeting malaria in pregnancy in malaria endemic areas should help clarify the specific contribution of this parasite in causing maternal morbidity and death.

The occurrence of co-morbidities has been associated with higher morbidity of malaria in different African locations, starting with HIV infection but also including other viral and bacterial co-infections [51-54], stressing the importance of investigating their occurrence and possibly promoting joint management strategies for common concurrent conditions in many tropical areas [55]. Bacterial systemic concurrent infections were rarely observed in Manaus, which could be a potential reflection that among adult patients the concomitant occurrence of bacterial infection seems much rarer than that among *P. falciparum*-infected children [56]. Yet other studies have reported similar rates affecting both age groups [57], in which factors associated with each setting could be playing a higher role, a topic we could not explore due to the systematic use of antibiotics in Bikaner. There were, however, important differences in the prevalence of co-morbidities in Manaus and Bikaner, with the former presenting considerably higher frequencies of both acute and chronic illnesses. The difference in the observed proportions of co-morbidities between malaria endemic areas is a reflection of the local epidemiology influenced by the demographic and socioeconomic structures, and also of the health systems’ capacities to diagnose and detect concomitant illnesses. Previous studies from the Brazilian Amazon region had already described the joint occurrence of malaria simultaneously with other health conditions, either acting as a contributing factor for severe manifestation or eventually being an incidental finding in patients with other severe diseases leading to critical illness or even fatal outcomes [14,25,58]. In a scenario where many tropical regions are facing epidemiological transition, the occurrence of *P. vivax* infection in individuals with chronic diseases is likely to become an important problem in these areas [59-61], putting a great deal of stress on and requiring effective recognition and management by local health systems.

We have been able to characterize a diverse range of clinical complications among the included patients, including a higher risk for death among the patients developing ARDS, which was more frequent in patients who had started antimalarials prior to hospitalization, similar to previously described cases [12,62]. Other clinical syndromes were also observed to occur with considerable frequency, noteworthy anemia requiring transfusion and acute renal failure, with cerebral malaria remaining a rare and intriguing manifestation within this infection [45]. The WHO severe malaria criteria were initially developed to identify individuals with *P. falciparum* infection at higher risk of death [63], and there have been discussions among experts on the need to define specific severity criteria to be applied for *P. vivax* infection. Our study demonstrates that the application of the WHO criteria can reliably identify the patients at higher risk of complications, who therefore require more urgent and intensive care, which further agrees with previous pediatric data from the same research group [15]. A comprehensive analysis of the array of complications observed among the patients in our study and in the published literature provides reassurance of these findings by demonstrating that the spectrum of clinical manifestations is broadly covered by these definitions [12,25], with unusual manifestations being rarely reported. However, it seems important to highlight that severity derived from the use of primaquine among G6PD-deficient individuals, or splenic rupture, two well-known complications mostly seen in vivax infections [58,64], are not included in the current WHO definitions and should be carefully considered by clinicians working in vivax endemic areas. Furthermore, one must consider that in primary and community healthcare units, the characterization of the fulfillment of all WHO criteria is not possible due to the unavailability of laboratory facilities. More easily applicable guidelines to identify severely ill patients regardless of etiology must be used by health professionals to assist with decisions on referral or more aggressive management.

Although it is not currently possible to reliably estimate the total parasite biomass in *P. vivax* infection due to the difficulty of assessing the extent of cytoadhesion [65] and spleen sequestration [66], we were able to observe that parasite densities within the higher quartile were associated with higher risk of severity. Among the clinical and laboratory markers, only total bilirubin presented a high discriminative performance to identify more than three criteria of severe disease, showing that although jaundice was justifiably excluded from the WHO severe malaria criteria, it still has an important prognostic value and should not be dismissed by health professionals in their initial assessment of *P. vivax*-infected patients. Intense abdominal pain and low hemoglobin levels should prompt health professionals to consider the diagnosis of spleen rupture or infarction and take the appropriate diagnostic and management procedures [64].

Our study has some clear limitations that need to be addressed. We decided to focus on providing a more comprehensive description of the clinical manifestations and complications associated with *P. vivax* infection in detriment to comparing disease expression between sites due to lack of adequate power and heterogeneity of procedures, undermining what could have been an important output from this study. The unrepresentativeness of children at both sites probably reflects the fact that neither of the sites is a reference center for pediatric populations, albeit publications from both locations have previously described severe manifestation among *P. vivax*-infected children [15,16]. The fact that the decisions to admit patients were made at the attending physician’s discretion may have resulted in added selection bias, which we tried to minimize by applying the WHO severe malaria criteria and determining the occurrence of more than three criteria (as a surrogate for more severe disease). Note also that there are important differences in the health system organization as well as particular disparities in the management of cases, such as for example the systematic use of antibiotics upon admission in India. Ensuring that both sites followed the common protocol proved also to be challenging, and in some cases probably hinders some of the comparisons in this study, especially regarding the presence of co-morbidities, such as malnutrition and other co-infections. Also, by not systematically measuring some immune molecules that have been associated with clinical complications of this infection, such as superoxide dismutase-1 [67], an opportunity was missed to properly evaluate the prognostic value of some promising biomarkers that could be of potential utility for future case management.

## Conclusions

This study has provided robust evidence asserting the role of *P. vivax* as a cause of severe human disease and death. Indeed, this infection commonly progresses with severe manifestations, and the development of severe symptomatology seems to be more frequent among females, pregnant women, individuals presenting with their first malarial infection, and those with acute or chronic co-morbidities. Although the overall the overall fatality rates are in concordance with findings from other *P. vivax* endemic areas, we observed differences between sites on specific disease manifestations and outcomes, which still require further and more comprehensive studies to be conducted to better elucidate the mechanisms and factors influencing disease expression. Only by understanding the underlying pathophysiological mechanisms by which this species initiates and modifies organ functions, ultimately leading to clinical disease, as well as the role of the socioeconomic and health systems, will we be able to start answering the many pending questions related to this never-to-be-called-again benign parasite.

## Additional file

Additional file 1: Table S1. Demographic and clinical characteristics of patients admitted with P. vivax infection that presented fatal outcome.
