# Supplementary material for: Characterization of Plasmodium vivax-associated admissions to reference hospitals in Brazil and India
Source: BMC Med. 2015 Mar 20;13:57. doi: 10.1186/s12916-015-0302-y (PMC4404636; doi:10.1186/s12916-015-0302-y)
Supplement: Additional file 1: Table S1. — Demographic and clinical characteristics of patients admitted with P. vivax infection that presented fatal outcome. [file 12916_2015_302_MOESM1_ESM.docx]

Supplementary Table. Demographic and clinical characteristics of patients admitted with P. vivax infection that presented fatal outcome

| Gender | Parasite density at arrival (parasites/mm^3^) | Previous malaria infection | Days of disease previous to admission | Number of severe WHO criteria | Previous  Comorbidities / Concomitant Conditions | Days from admission to death | Presumed mechanism of death |
| --- | --- | --- | --- | --- | --- | --- | --- |
| Manaus, Brazil | |  |  |  |  |  |  |
| M | Negative * | Yes | 9 | 2 | No | 0.5 | SA and ARF |
| F | 41251.6 | Yes | 3 | 9 | Hypertension | 14 | Subarachnoid hemorrhage |
| F | 995.2 | Yes | 3 | 12 | Pneumonia | 1 | MODS |
| Bikaner, India |  |  |  |  |  |  |  |
| F | 4850.2 | No | 10 | 9 | No | 6 | MODS |
| M | 22140.0 | No | 5 | 5 | No | 2 | ARF, MODS |
| F | 11537.8 | No | 4 | 4 | Pregnant | 6 | ARDS, ARF |
| F | 804.3 | No | 4 | 10 | Pregnant | 19 | ARDS, ARF |
| F | 11443.6 | No | 4 | 4 | Pregnant | 1 | ARDS, CM |
| F | 1633.8 | No | 5 | 8 | Pregnant | 10 | ARDS, CM |
| F | 8318.0 | No | 5 | 4 | No | 0 | ARDS, MODS |
| The age range of patients was 21-75 years of age (individual data suppress to preserve anonymity); * Received antimalarial previous to admission; SA- severe anemia; MODS – multiple organ dysfunction syndrome; ARF – acute renal failure; CM – cerebral malaria | | | | | | | |
